# The Pyroptosis-Related Gene Prognostic Index Associated with Tumor Immune Infiltration for Pancreatic Cancer

**DOI:** 10.3390/ijms23116178

**Published:** 2022-05-31

**Authors:** Wen Xie, Xiaoyi Li, Chunxiu Yang, Jiahao Li, Guoyan Shen, Hongshan Chen, Shu-Yuan Xiao, Yueying Li

**Affiliations:** 1 Department of Pathology, Wuhan University Zhongnan Hospital, Wuhan 430000, China; 2020103030001@whu.edu.cn (W.X.); circle_lxy@163.com (X.L.); cxyoung@whu.edu.cn (C.Y.); lijiahao@whu.edu.cn (J.L.); 15071406231@163.com (G.S.); hongshan_26@163.com (H.C.); 2 Wuhan University Center for Pathology and Molecular Diagnostics, Wuhan 430000, China; 3 Department of Pathology, University of Chicago Medicine, Chicago, IL 60637, USA

**Keywords:** pancreatic cancer, pyroptosis-related gene, prognostic index, immune infiltration, therapy

## Abstract

Pancreatic cancer (PC) is one of the most fatal malignancies. Pyroptosis, a type of inflammatory cell death, likely plays a critical role in the development and progression of tumors. However, the relationship between pyroptosis-related genes (PRGs) and prognosis and immunity to PC is not entirely clear. This study, aimed at identifying the key PRGs in PC, highlights their prognostic value, immune characteristics, and candidate drugs for therapies. We screened 47 differentially expressed PRGs between PC and normal pancreas tissues from The Cancer Genome Atlas (TCGA) and Genotype-Tissue Expression (GTEx) datasets. Afterwards, a pyroptosis-related gene prognostic index (PRGPI) was constructed based on eight PRGs (AIM2, GBP1, HMGB1, IL18, IRF6, NEK7, NLRP1 and PLCG1) selected by univariate and multivariate Cox regression analysis and LASSO regression analysis, and verified in two external datasets from the International Cancer Genome Consortium (ICGC) and Gene Expression Omnibus (GEO) databases. We found that the PC patients in the PRGPI-defined subgroups not only reflected significantly different levels of infiltration in a variety of immune cells, such as M1 macrophages, but also showed differential expression in genes of the human leukocyte antigen (HLA) family and immune checkpoints. Additionally, molecular characteristics and drug sensitivity also stayed close to the PRGPI risk scores. Therefore, PRGPI may serve as a valuable prognostic biomarker and may potentially provide guidance toward novel therapeutic options for PC patients.

## 1. Introduction

Pancreatic cancer (PC) is the fourth most lethiferous cancer in the United States, with an estimated 57,600 new cases and 47,050 deaths occurring annually in 2019 [1]. In China, PC is also the sixth leading cause of cancer-related death, with an increasing tendency toward incidences and mortalities [2]. It is expected that PC will become the second leading cause of cancer mortality by 2030 worldwide [3]. PC lacks effective therapies other than surgical resection, but either unresectable or metastatic disease was found in 80–85% of patients at the time of diagnosis, leading to extremely poor overall survival [4,5]. The high mortality associated with PC is also attributed to the complex tumor microenvironment (TME) and drug resistance to the current treatment [6,7,8]. Thus, there is an urgent need to establish better diagnostic and prognostic markers and more effective therapeutic options for PC.

Pyroptosis, also known as cellular inflammatory necrosis, is a type of inflammasome-induced programmed cell death, which is featured by the continuous enlargement of cells until the cell membrane ruptures, resulting in the release of cellular contents (including inflammatory cytokines) and an intense inflammatory response [9,10,11]. The occurrence of pyroptosis depends on the caspase and gasdermins (GSDMs) protein family, that is, activated caspases cleave GSDMs and release its N-terminal domain, which binds to membrane lipids to form pores, leading to changes in cellular osmotic pressure, causing swelling of the cell until the membrane ruptures [9,12,13,14]. The GSDMs protein family, as a key effector molecule of pyroptosis, is known to be encoded by six paralogous genes: *GSDMA*, *GSDMB*, *GSDMC*, *GSDMD*, *GSDME* (also known as *DFNA5*) and *DFNB59* (also known as *PJVK*) [15]. It is found that GSDMD can be activated by caspase-1/4/5/11 [16,17,18], GSDME by caspase-3 [19], and GSDMB by caspase-3/6/7 [20]. At present, the best-characterized pathway of pyroptosis is relatively clear: caspase-1 is activated within multi-protein inflammasome complexes that assemble in response to invasive infection and red flags, which leads to the cleavage of GSDMD, accompanied by the maturation and secretion of a large number of pro-inflammatory cytokines, such as IL-18 and IL-1β [9].

The mechanism and functions of pyroptosis in cancer is complex. On the one hand, pyroptosis inhibits the oncogenesis and progression of tumors; on the other hand, it can help develop a suitable tumor microenvironment and thus accelerate tumor cell growth [21,22,23]. Additionally, inflammation induced by pyroptosis can trigger powerful antitumor immunity and can work synergistically with a checkpoint blockade [24,25]. It has been shown that pyroptosis induced by caspase-1 can suppress the proliferation, migration, invasion, and cell spheroid formation of PC cells [26]. Some pyroptosis-related genes (PRGs) may be valuable research targets for PC, and their expression can be combined with specific immune characteristics to predict the survival benefit to patients [27,28]. However, researchers have speculated that pyroptosis can also play a dual role in the progression and therapeutic response of PC, but studies of pyroptosis in PC are scarce [29].

**Figure 1:**
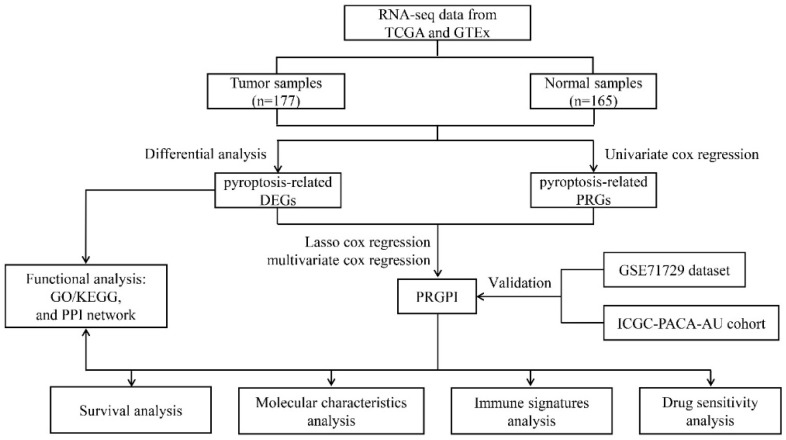
Flowchart of the overall procedures. The process of data collection and analyses for prognostic studies are illustrated.

In the current study, we focused on the key PRGs and identified differentially expressed genes (DEGs) in these PRGs between PC tissues and normal pancreas tissues from the public datasets. Then, we constructed a pyroptosis-related gene prognostic index (PRGPI) based on the optimal PRGs related to prognosis screened by univariate and multivariate Cox regression analysis and LASSO regression analysis, and verified in two external datasets. Finally, prognostic value, functional enrichment, molecular and immune characteristics, and drug sensitivity in PRGPI-defined subgroups were analyzed. The flow chart of this study is shown in Figure 1.

## 2. Results

### 2.1. Identification and Functional Enrichment Analysis of Pyroptosis-Related DEGs

**Figure 2:**
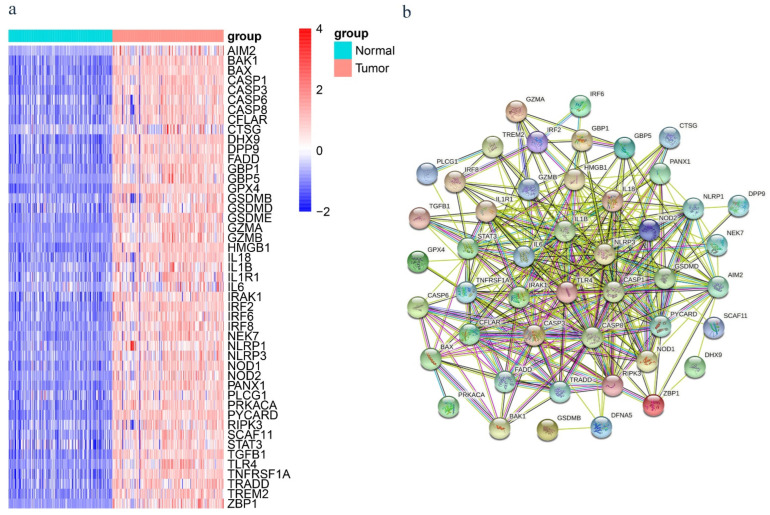
(**a**) Heatmap of the 47 pyroptosis-related differentially expressed genes (DEGs) in normal and tumor tissues from the GTEx and TCGA datasets. (**b**) PPI network showing the interactions of the pyroptosis-related DEGs (interaction score = 0.4).

In differential expression analysis (177 tumors versus 165 normal tissues), a total of 8952 DEGs were obtained, of which 8493 genes were upregulated and 469 genes were downregulated in the tumor tissues compared to normal tissues (Supplementary Figure S1a–c). By intersecting these genes with the lists of PRGs, 47 differentially expressed PRGs were obtained, and all of them were upregulated in tumor tissues compared with normal tissues (Figure 2a). The functional enrichment analysis was performed based on 47 pyroptosis-related DEGs, and the top 10 GO-BP/MF/CC terms and KEGG pathways are shown in Supplementary Figure S2a–d. To further explore the interactions of these PRGs, a PPI analysis was conducted, and the results are shown in Figure 2b.

### 2.2. Construction of PRGPI

**Figure 3:**
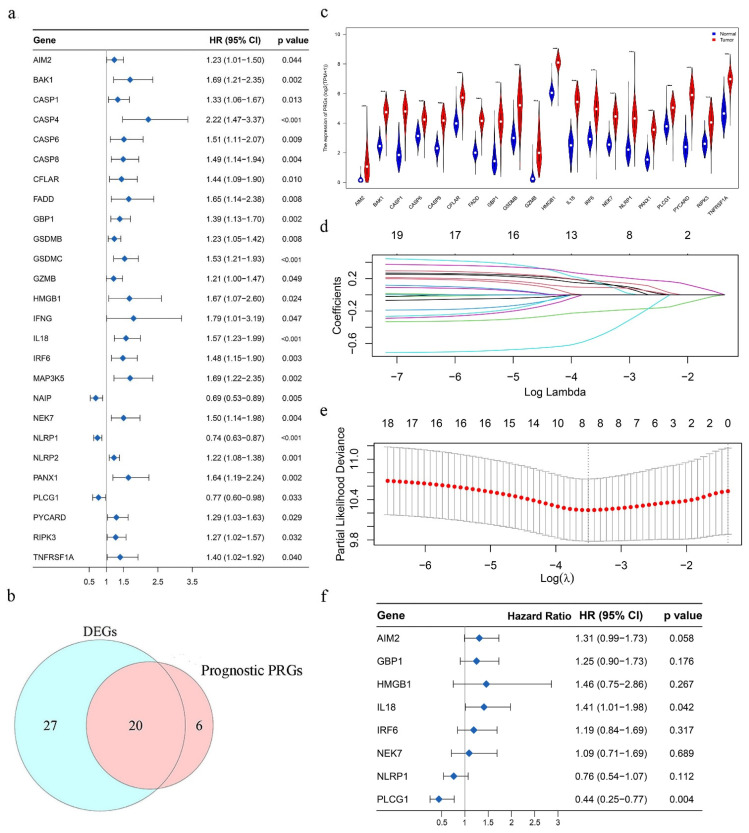
(**a**) Forest plot showing the results of univariate Cox regression analysis between the expression and prognosis of 26 pyroptosis-related genes (PRGs). (**b**) Venn plot to identify the 20 pyroptosis-related DEGs that were correlated with OS. (**c**) Expression of 20 PRGs in pancreatic cancer (PC) and normal pancreas tissues. *p* value < 0.001. (**d**) LASSO regression of the 20 prognostic PRGs. (**e**) Cross-validation for tuning parameter selection in the LASSO regression. (**f**) Forest plot to show the results of the multivariate Cox regression analysis between 8 optimal PRGs’ expression and prognosis.

First, 26 prognosis-related genes were screened from all 72 PRGs by the univariate Cox regression analysis of OS (*p* < 0.05) (Figure 3a). Twenty of them were also pyroptosis-related DEGs (Figure 3b), and the differential expression of these genes in PC and normal pancreatic tissue is shown in the violin plot (Figure 3c). To prevent the risk of over-fitting, the LASSO regression analysis was utilized to narrow down the candidate genes based on the minimum value of lambda λ (Figure 3d,e). The corresponding coefficient of the optimal eight PRGs were obtained by multivariate Cox regression analysis (Figure 3f). Then, we constructed a prognostic index for all PC individuals calculated by the formula PRGPI risk score = 0.2688 × expression of *AIM2* + 0.2244 × expression of *GBP1* + 0.3797 × expression of *HMGB1* + 0.3468 × expression of *IL18* + 0.1764 × expression of *IRF6* + 0.0820 × expression of *NEK7* + (−0.2797) × expression of *NLRP1* + (−0.8197) × expression of *PLCG1*.

### 2.3. Evaluation and Validation of PRGPI

**Figure 4:**
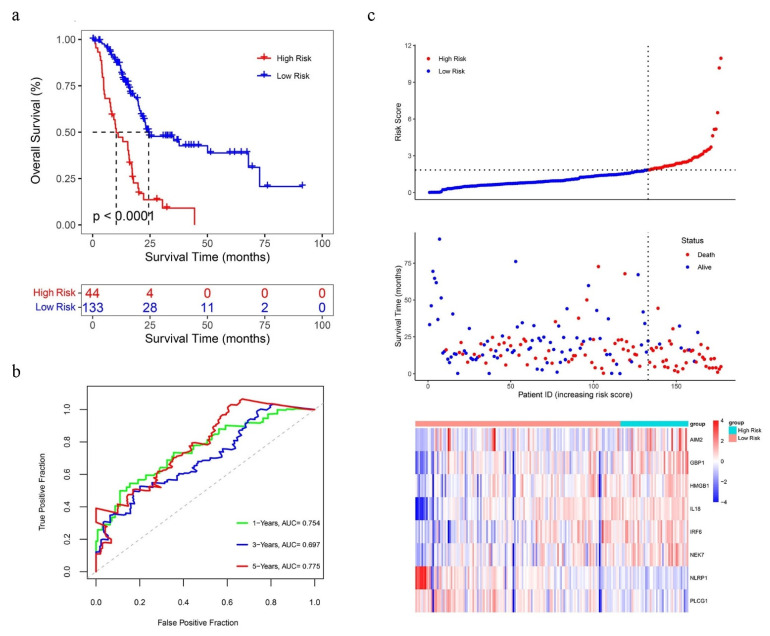
(**a**) Kaplan–Meier curves for OS of PC patients in the high/low pyroptosis-related gene prognostic index (PRGPI) risk groups. (**b**) Receiver operating curves (ROC) curves demonstrating predictive efficiency of the PRGPI risk score. (**c**) Distribution of risk score, survival status, and the expression of eight prognostic PRGs in PC.

According to the best cut-off value of PRGPI risk score, patients with PC in all three datasets were divided into low and high PRGPI risk groups. The survival curves showed that the high PRGPI risk had a lower probability of survival in the TCGA dataset (Figure 4a, *p* < 0.0001). The predictive effects of the PRGPI risk scores for OS were shown by the ROC curves, and the AUC of the PRGPI reached 0.754 (1-year), 0.697 (3-year), and 0.775 (5-year), indicating a strong separation capability (Figure 4b). The scatterplots (Figure 4c top) show the distribution of PRGPI risk scores and the correlation between PRGPI risk scores and OS in the TCGA dataset. The high PRGPI risk patients were more likely died earlier than the low PRGPI risk patients (Figure 4c middle). The heatmap (Figure 4c bottom) shows the prognostic risk gene-expression profiles between the low and high PRGPI risk groups in the TCGA dataset.

**Figure 5:**
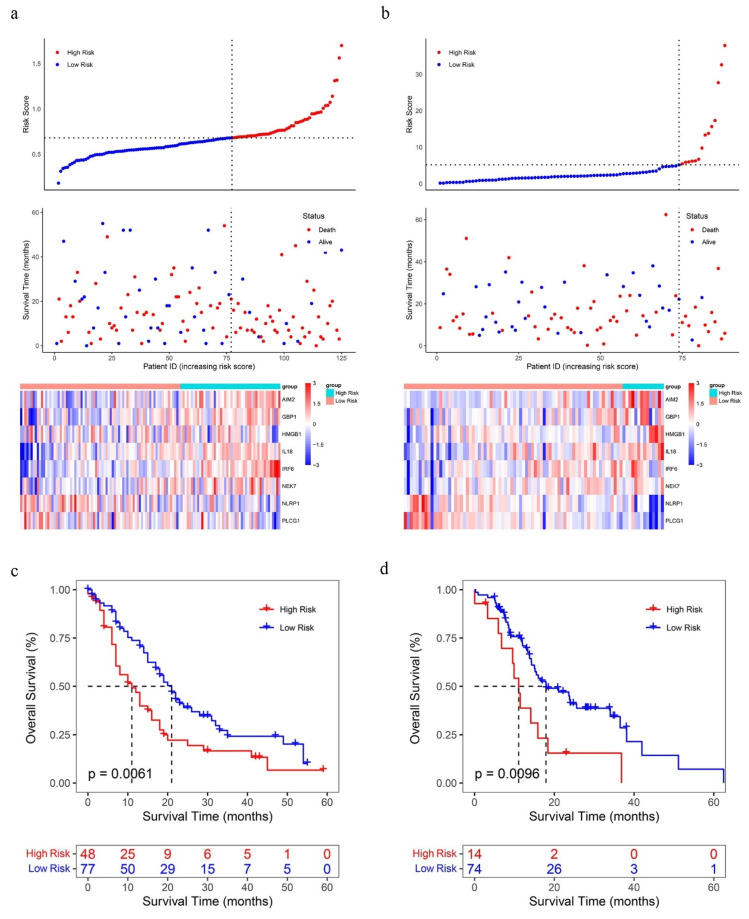
The OS status plots, OS and risk score plots and heatmaps of these 8 genes in the GSE71729 dataset (**a**) and ICGC-PACA-AU dataset (**b**), respectively. Kaplan–Meier curves for the OS of PC patients in the high/low PRGPI risk group of GSE71729 dataset (**c**) and ICGC-PACA-AU dataset (**d**).

The same results were also verified in the ICGC and GSE71729 datasets (Figure 5a,b). Furthermore, In the ICGC and GSE71729 datasets, low PRGPI risk patients had a higher probability to have a prolonged OS time compared with high PRGPI risk patients (Figure 5c,d, *p* < 0.05).

### 2.4. Analysis of Independent Prognostic Factors and Construction and Validation of Nomogram

**Figure 6:**
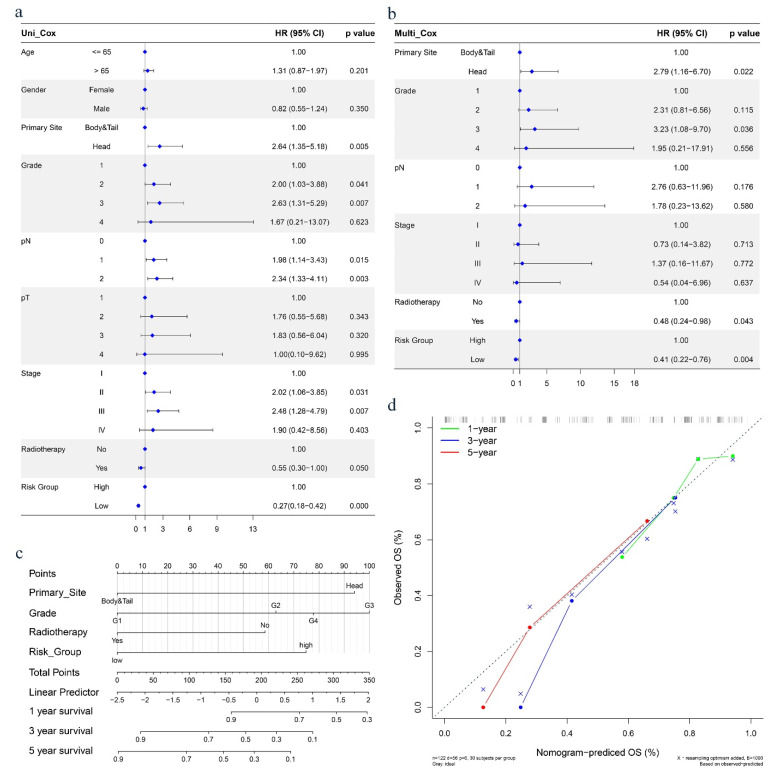
(**a**,**b**) Results of the univariate and multivariate Cox regression analyses regarding OS in the TCGA dataset. (**c**) Nomograms for predicting 1-, 3-, and 5-year survival in the TCGA dataset. (**d**) Calibration curves for the nomogram predicting 1-, 3-, and 5-year survival in the TCGA dataset.

The univariate Cox regression analysis revealed that the PRGPI risk group (*p* < 0.001) and related clinical characteristics, including tumor primary site, grade, lymph node status (pN), TNM stage and radiotherapy (*p* < 0.05) were significantly associated with OS in the TCGA dataset (Figure 6a). Multivariate Cox regression analysis indicated that the PRGPI risk group, primary site, grade and radiotherapy (*p* < 0.05) were the independent prognostic factors of the OS (Figure 6b). The clinical characteristics including age, gender, T, N, stage, and PRGPI risk grouping were included to construct the nomogram prediction models in the TCGA dataset (Figure 6c) to show the survival prediction for PC patients at 1, 3, and 5 years. The calibration curves were shown to indicate great prediction in the actual observations in 1, 3, and 5 years (Figure 6d).

### 2.5. Functional Enrichment and Molecular Characteristics Based on the PRGPI

The GO enrichment and KEGG pathway analysis based on DEGs between the high and low PRGPI risk groups were performed to explore the functions and pathways that were related to the PRGPI risk score. The results indicated that the DEGs were mainly correlated with the bacterial infection, apoptosis, cell cycle, RNA catabolic process, focal adhesion, cell-substrate junction, cadherin binding and ubiquitin-like protein ligase binding (Supplementary Figure S3a–d).

**Figure 7:**
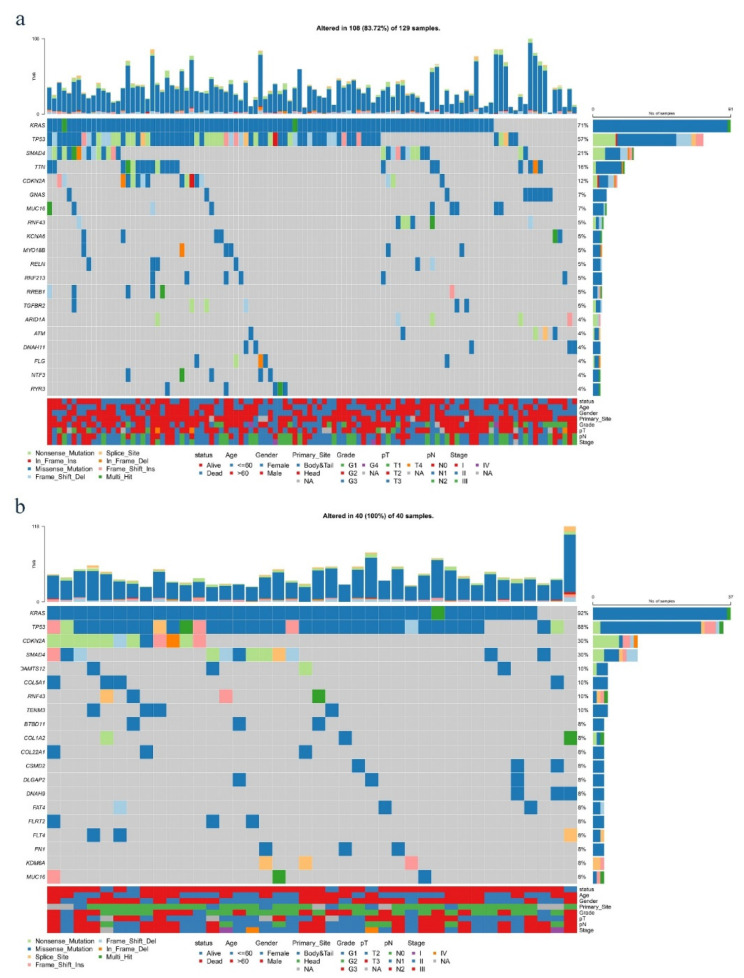
The oncoplots of the somatic mutation between the low PRGPI risk (**a**) and high PRGPI risk (**b**) groups in the TCGA dataset.

Next, we analyzed gene mutations in the TCGA dataset to gain further biological insight into the molecular characteristics of the PRGPI risk subgroups. The summaries of the gene mutation information are shown in the bar plot (Supplementary Figure S4a,b). We found that *KRAS* (92%), *TP53* (88%), *SMAD4* (30%), *CDKN2A* (30%) and *DAMTS12* (10%) were the top five genes with the highest mutation frequencies in the high PRGPI risk group, and *KRAS* (71%), *TP53* (57%), *SMAD4* (21%), *TTN* (16%) and *CDKN2A* (12%) in the low PRGPI risk group (Figure 7a,b).

### 2.6. PRGPI Was Associated with Immune Signatures of PC

**Figure 8:**
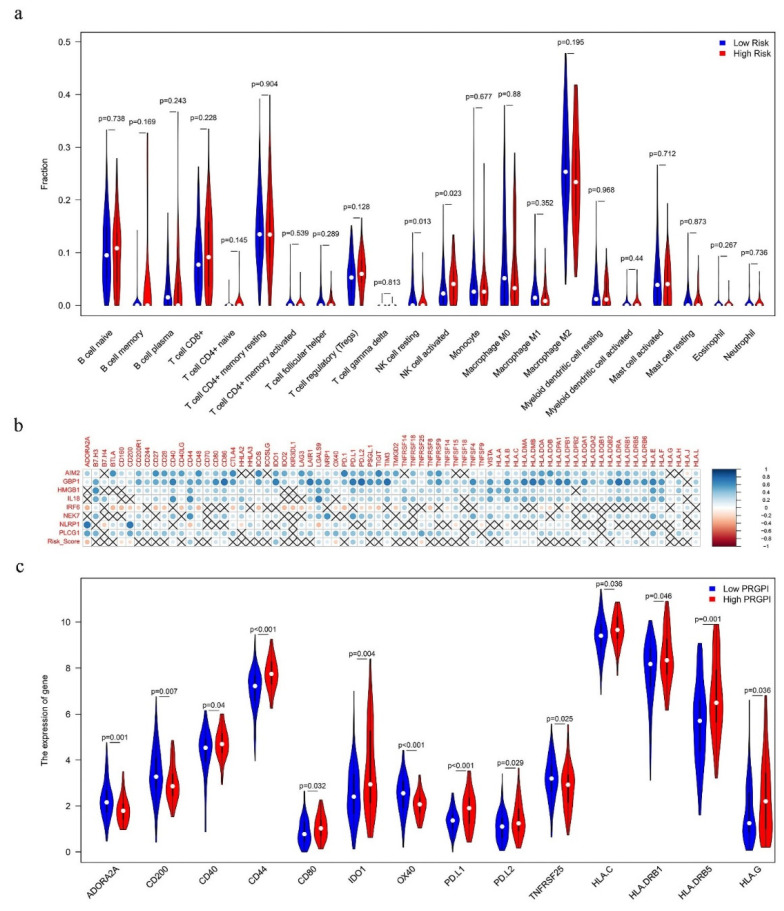
(**a**) Comparison of immune cell infiltration between the low and high PRGPI risk groups of the TCGA dataset. (**b**) Correlation analysis for PRGPI risk score and expression of human leukocyte antigen (HLA) family genes/immune checkpoints. (**c**) Analysis of expression of immune checkpoints and HLA family genes in different PRGPI risk groups.

To better study how the PRGPI interacts with the immune microenvironment, we used the CIBERSORT algorithm to evaluate the proportion of tumor-infiltrating immune subpopulations and made comprehensive comparisons with the PRGPI risk scores. Incorporating the results of difference analysis, activated mast cells, M1 macrophages, and plasma cells were identified, associated with different PRGPI risk groups (Figure 8a). The relative content distribution of 22 TICs in the TCGA dataset are shown in Supplementary Figure S5a,b.

Additionally, we also studied the gene expression of the 24 HLA family genes and 48 immune checkpoints between the high and low PRGPI risk groups. We found that the PRGPI risk score was significantly correlated with the expression of 16 HLA genes and 28 immune checkpoints, including *PD-L1*, *PD-L2*, *IDO1*, *CD40* and *HLA-B*, etc. (Figure 8b). In addition, four HLA family genes and 10 immune checkpoints were significantly modulated in the PRGPI risk groups (Figure 8c). 

### 2.7. PRGPI May Predict Chemotherapeutics Response

**Figure 9:**
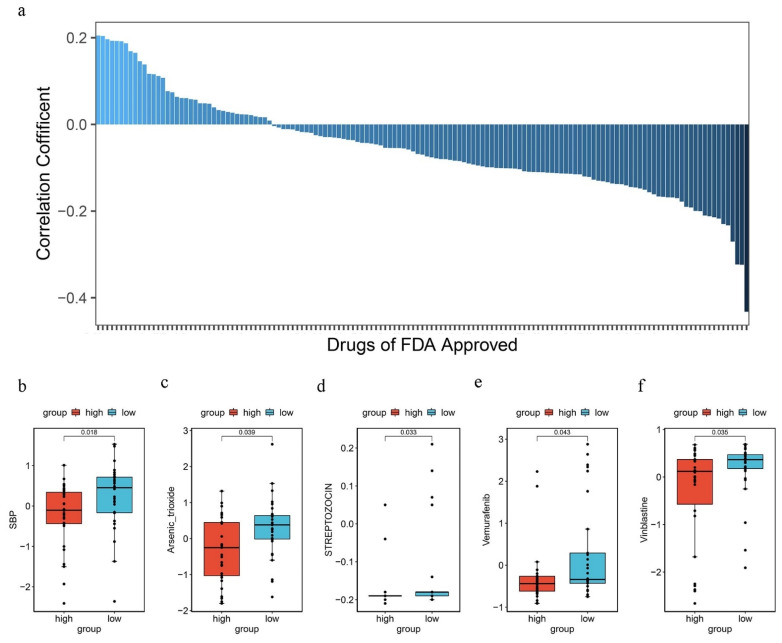
(**a**) Respective 50% inhibiting concentration (IC50) value of chosen compounds in relation to the PRGPI risk score, as shown by Spearman correlation analysis. (**b**–**f**) Those with high PRGPI risk scores were found to possess lower IC50 scores for FDA-approved chemotherapeutics, such as 6-Mercaptopurine, Arsenic trioxide, Streptozocin, Vemurafenib, and Vinblastine.

To study whether the PRGPI can predict chemosensitivity, the PRGPI risk scores of NCI60 cell lines were calculated from the expression data available in a CellMiner database. After calculating the correlation between the PRGPI risk score and the IC50 value of 218 FDA-approved drugs across 60 cell lines, it was found that Cisplatin, Temsirolimus, Rapamycin and Everolimus appeared to be significant with the risk model (|Spearman correlation| > 0.2 and *p* < 0.05, Figure 9a). A high immune score was associated with a lower IC50 of medications including 6-Mercaptopurine, Arsenic trioxide, Streptozocin, Vemurafenib, and Vinblastine (Figure 9b–f, *p* < 0.05). These findings indicate that the PRGPI can play a role as a chemosensitivity predictor. 

## 3. Discussion

PC is a malignant gastrointestinal tumor with high aggressiveness and mortality. Despite efforts made to develop more effective therapeutic strategies, efforts to improve chances for survival for patients with PC still show very limited results, and only offer marginal benefits [7,8,30]. In view of this, it is necessary to explore the changes in the expression and mechanisms of key genes in PC from the perspective of clinical therapy and prognosis, in order to identify better biomarkers and fully understand the underlying features of PC. Pyrolysis is a double-edged sword, affecting the biology and therapeutic response of tumors. On one hand, working with local or systemic treatments, it may inhibit cancer proliferation, invasion and metastasis; on the other hand, unrestricted pyrolysis or inflammation may lead to an immunosuppressive microenvironment, which contributes to tumor progression or recurrence [24,25]. As the specific role of pyroptosis in PC is unclear, the most straightforward and concrete way is to investigate the clinical significance of PRGs in the prognostic prediction and therapeutic benefits of PC.

In this study, we have systematically investigated the mRNA expression levels of PRGs in samples from the TCGA and GTEx datasets, and found that most of the genes (65.28%, 47/72) were significantly differentially expressed between the PC and normal tissue, suggesting that pyroptosis plays an important role in PC pathogenesis. Compared to normal tissue, the expression of all DEGs in PC was upregulated, 20 of which were further confirmed to have prognostic values by univariate Cox regression analysis. Using LASSO regression analysis and multivariate Cox regression analysis, a novel PRGPI based on the optimal eight PRGs was constructed and externally validated in two external datasets (ICGC and GSE71729), including risk genes (*AIM2*, *GBP1*, *HMGB1*, *IL18*, *IRF6* and *NEK7*) and protective genes (*NLRP1* and *PLCG1*). It was found that PC patients in the low-risk group had a significantly longer OS, not only in the TCGA dataset, but the prognostic results of PRGPI were also found in the two external validated datasets from the ICGC and GEO databases.

AIM2, as a cytosolic double-stranded DNA (dsDNA) sensor that induces caspase-1-dependent IL-1β maturation, is one of the components of the AIM2 inflammasome that induces pyrolysis [31]. Studies have shown that the AIM2 inflammasome plays an oncogenic-like role in pancreatic tumorigenesis and high AIM2 expression is associated with poor prognosis in patients with PC [32]. However, in colorectal cancer, the inflammasome-independent effect of AIM2, primarily mediated by a non-bone marrow source of AIM2, may result in the suppression of colon tumorigenesis [33]. GBP1 acts a gatekeeper of microbe-induced macrophage apoptosis and pyroptosis, and IFNγ enhanced macrophage pyroptosis mediated by caspase-4 in a GBP1-dependent manner during bacterial infection, such as Shigella flexneri and Salmonella [34,35]. HMGB1 secreted by hepatocytes has been reported to deliver extracellular LPS into the cytoplasm and mediate caspase-11-dependent pyroptosis [36]. Meanwhile, oxidized HMGB1 may trigger CD274/PD-L1 expression in an AGER-dependent manner, thereby allowing tumors to evade anticancer immunosurveillance in PC cells [32]. IL-18, as a member of the IL-1 family of cytokines, is involved in the activation of T cells, NK cells and macrophages, and plays an important role in the process of immunoregulation in various inflammatory and malignant diseases, including pyroptosis and cancer [37]. At present, it is known that free IL-18 in the serum is a poor prognostic marker for PC patients, and studies have speculated that IL-18 has a dual effect on tumor cells: to promote growth and expansion of PC cells by activation of the NF-κB signaling pathway, and to inhibit PC growth through activation of the anti-tumor immune response [38,39]. IRF6 is a transcription factor in the regulation of the host defense and is crucial for stimulating tumorigenesis [40,41]. According to reports, NEK7 is an important component of the NLRP3 inflammasome in macrophages that can regulate the occurrence of pyroptosis, can promote PC progression, and may be a potential marker for PC prognosis [42,43]. NLRP1 is the first pattern recognition receptor discovered to form an inflammasome, and it has been confirmed that when the C-terminal fragment is released, it can recruit and activate pro-CASP1 and induce pyroptosis [44]. PLCG1 is a class of membrane-associated enzymes involved in cell death and inflammatory reactions and contributes to GSDMD-N-mediated cytotoxicity in a calcium-dependent manner, which is an important intermediate process of pyroptosis [45,46]. All in all, the molecular functions of these genes are all related to inflammation, cancer or anti-tumor immunity. 

Through enrichment analysis for DEGs in the low and high PRGPI risk groups, we found that bacterial infection, apoptosis, cell cycle, RNA catabolic process, focal adhesion, cell-substrate junction, cadherin binding, and ubiquitin-like protein ligase binding were enriched. Pyrolysis can be triggered by a variety of in vitro pathogens, but it is also the main mechanism for eliminating bacteria in cells [47,48,49]. In addition, some genes in pyroptosis are also known as key regulators in apoptotic pathways, such as GBP1, HMGB1, and PLCG1 [10,50]. Based on the above results, we have reason to suspect that pyroptosis can regulate development and metastasis of PC as expected. In addition, to further characterize molecular profiles for the PRGPI risk subgroups, we studied gene mutations in the low and high PRGPI risk groups. We found that missense variations were the most common, followed by nonsense and frameshift deletions, which is consistent with previous reports [51]. More importantly, compared to the low PRGPI risk group, the high PRGPI risk group has a higher mutation frequency of *KRAS* (92% vs. 71%), *TP53* (88% vs. 57%), *SMAD4* (30% vs. 21%) and *CDKN2A* (30% vs. 12%). As is known, the development of PC is a process characterized by the accumulation of gene mutations, and the accompanying aberrant genetic events are generally observed in oncogene *KRAS* (activation) and tumor suppressors *TP53*, *CDKN2A* and *SMAD4* (inactivation) [52]. Studies have shown that patient outcomes are associated with alterations of these four main driver genes in resected PC and patients with mutated genes have significantly shorter OS than patients with wild-type genes [53]. This is consistent with our survival results, and to a certain extent also shows that the prognostic value of the PRGPI we constructed is effective. 

Another finding from this study is the difference in composition of immune cells between the two PRGPI risk groups. While plasma cells and M1 macrophages were more enriched in the high PRGPI risk group, activated mast cells were more common in the low PRGPI risk group. In general, M1 macrophages are involved in inflammation, pathogen clearance and anti-tumor immunity, and a high density of M1 macrophages may mean a better prognosis for patients with cancer [54,55]. Although this contradicts our conclusion, other studies have shown that M1 macrophages may promote the occurrence of tumors and are related to poor prognosis [56,57]. Therefore, we speculate that the simple use of batch sequencing in tissues to estimate immune infiltration and judge tumor immune signatures may not be sufficiently accurate. Further studies on the relationship between PRGs and tumor-associated macrophages (TAMs) in PC and their correlation with the prognosis of patients are worthy of future research. Our results also showed that the high PRGPI risk group tended to predict an immune suppressed status with the upregulated expression of most immune checkpoints and HLA family genes, such as *PD-L1*, *PD-L2* and *IDO1*. In the past decade, immune checkpoint inhibitor (ICI) therapy, as an emerging tumor treatment method, has shown a durable clinical benefit in a variety of malignancies [58,59]. PD-L1, also known as B7-H1, is one of the two ligands of programmed death receptor 1 (PD-1). Increased expression of PD-L1 on cancer cells or stroma cells is the basic mechanism for evading host immunity and is also related to the poor prognosis of PC patients [60]. Moreover, a previous study indicated that PC targeted for PD-L1 therapy did not achieve good clinical benefits and there was no response to monotherapy [61]. It can be said that due to the tumor’s complex, highly immunosuppressive microenvironment, the immunotherapy of PC still has a long way to go.

At the present, chemotherapy is still the main treatment for patients with advanced PC [4]. Therefore, we investigated whether the PRGPI could predict the chemosensitivity in PC. Our results showed that the IC50 values were negatively correlated with the PRGPI risk scores and were significantly higher in the low PRGPI risk group for some anticancer drugs. Among these drugs, cisplatin is a classic chemotherapy drug widely used in the treatment of solid tumors. Although the effect of cisplatin monotherapy in pancreatic cancer is not obvious, it may become an effective treatment plan when combined with gemcitabine, epirubicin and 5-FU [62].

## 4. Materials and Methods

### 4.1. The Datasets and Samples

Gene expression, somatic mutation and relevant clinical data of PC were searched for in The Cancer Genome Atlas (TCGA) database and gene expression data of normal pancreas tissues were searched for in the Genotype-Tissue Expression (GTEx) database. As to these datasets in TCGA and GTEx, all data were downloaded from the UCSC Xena Database (UCSC, http://xena.ucsc.edu/, accessed on 9 December 2021). Moreover, gene expression data and the corresponding clinical information of GSE71729 and ICGC- PACA-AU datasets were retrieved from the Gene Expression Omnibus (GEO, http://www.ncbi.nlm.nih.gov/geo, accessed on 12 December 2021) and International Cancer Genome Consortium (ICGC, https://dcc.icgc.org, accessed on 12 December 2021) databases, respectively. The systematical screening criteria were as follows: information of samples was relatively complete and substantial. Patient information from the TCGA dataset is presented in Table 1.

### 4.2. Identification of DEGs

Seventy-two PRGs were extracted from previous reviews [10,11,14], which are shown in Table S1. On the basis of the gene expression data of PC samples (177 tumors vs. 165 normal tissues) obtained from the TCGA and GTEx, lists of DEGs (*p* < 0.05, |log_2_FC| > 1) were identified by using the “limma” package of R software 4.1.2. To understand the possible function of pyroptosis-related DEGs in PC, the Gene Ontology (GO) function and Kyoto Encyclopedia of Genes and Genomes (KEGG) pathway enrichment analysis were performed using “clusterProfiler” R package based on these DEGs, and then visualized using “ggplot2” R package. GO function enrichment analysis included Biological Process (BP), Molecular Function (MF), and Cell Component (CC). Moreover, a protein-protein interaction (PPI) network for the DEGs was obtained from Search Tool for the Retrieval of Interacting Genes (STRING, https://string-db.org/, version 11.5, accessed on 10 December 2021).

### 4.3. Identification of Key Prognostic Genes and Establishment of the PRGPI

To determine the survival significance of PRGs (*p* < 0.05), we carried out univariate Cox regression analysis. Then, the candidate genes were narrowed by LASSO regression analysis and the penalty parameter (λ) was determined by the minimum parameters. Finally, we calculated the regression coefficients of these optimal FRGs and constructed an PRGPI by multivariate Cox regression analysis. The PRGPI risk score was calculated using the formula “(regression coefficients × corresponding mRNA expression)”. 

### 4.4. Prediction and Validation Analysis

The “survivalROC” package was used to generate receiver operating characteristics (ROC) curves at 1, 3, and 5 years, and the corresponding time-dependent area under the curves (AUCs) was calculated. In addition, all patients were classified into low and high PRGPI risk groups according to the best cut-off value of PRGPI risk score from the “survminer” package. The difference in prognosis between the two groups was confirmed by Kaplan–Meier (K-M) survival curve. Since external validation is critical for prognostic signatures, we used the GSE71729 and ICGC datasets to validate the prognostic value of PRGPI. 

### 4.5. Prognostic Value Assessment and Nomogram Prediction Model Construction

Univariate and multivariate Cox regression analyses were performed to assess whether the PRGPI risk grouping is related to prognosis of PC in the TCGA dataset. The “rms” R package was used to establish the nomogram of the TCGA dataset to predict the survival probability of PC individuals at 1, 3, and 5 years, and the calibration curves were drawn.

### 4.6. Functional Enrichment Analysis for DEGs between Two PRGPI Risk Subgroups

DEGs between the low and high PRGPI risk samples in the TCGA dataset were identified by the “limma” package of R. The “clusterProfiler” R package was used to perform function enrichment analysis of the DEGs with *p* < 0.05. At the same time, somatic mutation data of low and high PRGPI risk groups in the TCGA dataset were analyzed and visualized using the “maftools” R package. 

### 4.7. Analysis of Tumor Immune Signatures for PRGPI Risk Score

Gene expression data from the TCGA dataset was transformed into the total abundance of immune cells by utilizing the Cell-type Identification by Estimating Relative Subsets of RNA Transcripts (CIBERSORT, https://cibersort.stanford.edu/, accessed on 24 December 2021) analysis with the “CIBERSORT” R package. The association between the PRGPI risk score and the expression of the human leukocyte antigen (HLA) gene family and immune checkpoints was performed by the Wilcoxon rank-sum test and Spearman correlation analysis [63].

### 4.8. Assessment of the Clinical Drug Response Prediction of PRGPI Risk Score

We investigated the predictive capacity of PRGPI risk score in 218 drugs from chemotherapies/targeted therapies approved by the Food and Drug Administration (FDA). The gene expression profiles and drug sensitivity 50% inhibiting concentration (IC50) values of the NCI-60 panel of human cancer cell lines were extracted from the CellMiner database (https://discover.nci.nih.gov/cellminer/, accessed on 24 December 2021). The difference in the IC50 Z-score between the high and low PRGPI risk groups was analyzed using the Wilcoxon test. 

### 4.9. Statistical Analysis

All statistical analysis were performed using RStudio software 1.4.1717 and its appropriate packages. All *p* values of statistical data were based on two-sided statistical tests, and data with *p* < 0.05 was considered to be statistically significant.

## 5. Conclusions

PRGPI constructed based on PRGs is a good prognostic risk model related to tumor immune infiltration for PC patients. It may serve as a potential biomarker for better prediction of prognosis and may provide guidance toward new therapeutic options for PC patients. These findings may also bring us new insights into the underlying molecular mechanisms for PC and pyroptosis, but more studies are needed for further clarification. 

## Figures and Tables

**Table 1:** Clinicopathological characteristics of PC patients in the TCGA dataset.

Characteristics	Variable	TCGA Dataset (*n* = 177)
Age, years	≤65	94
	>65	83
Gender	Female	80
	Male	97
Grade	G1	30
	G2	95
	G3	48
	G4	2
	GX	2
Primary site	Head	131
	Body & Tail	32
	Unknown	14
pT	T1	10
	T2	96
	T3	55
	T4	3
	TX	13
pN	N0	51
	N1	73
	N2	52
	NX	1
TNM Stage	I	32
	II	83
	III	54
	IV	4
	Unknown	4
Radiotherapy	Yes	37
	No	101
	Unknown	39

## Data Availability

Not applicable.

## Supplementary Materials

The following supporting information can be downloaded at: www.mdpi.com/article/10.3390/ijms23116178/s1.
