# Supplementary figures and images for: The Pyroptosis-Related Gene Prognostic Index Associated with Tumor Immune Infiltration for Pancreatic Cancer
Source: Int J Mol Sci. 2022 May 31;23(11):6178. doi: 10.3390/ijms23116178 (PMC9180955; doi:10.3390/ijms23116178)

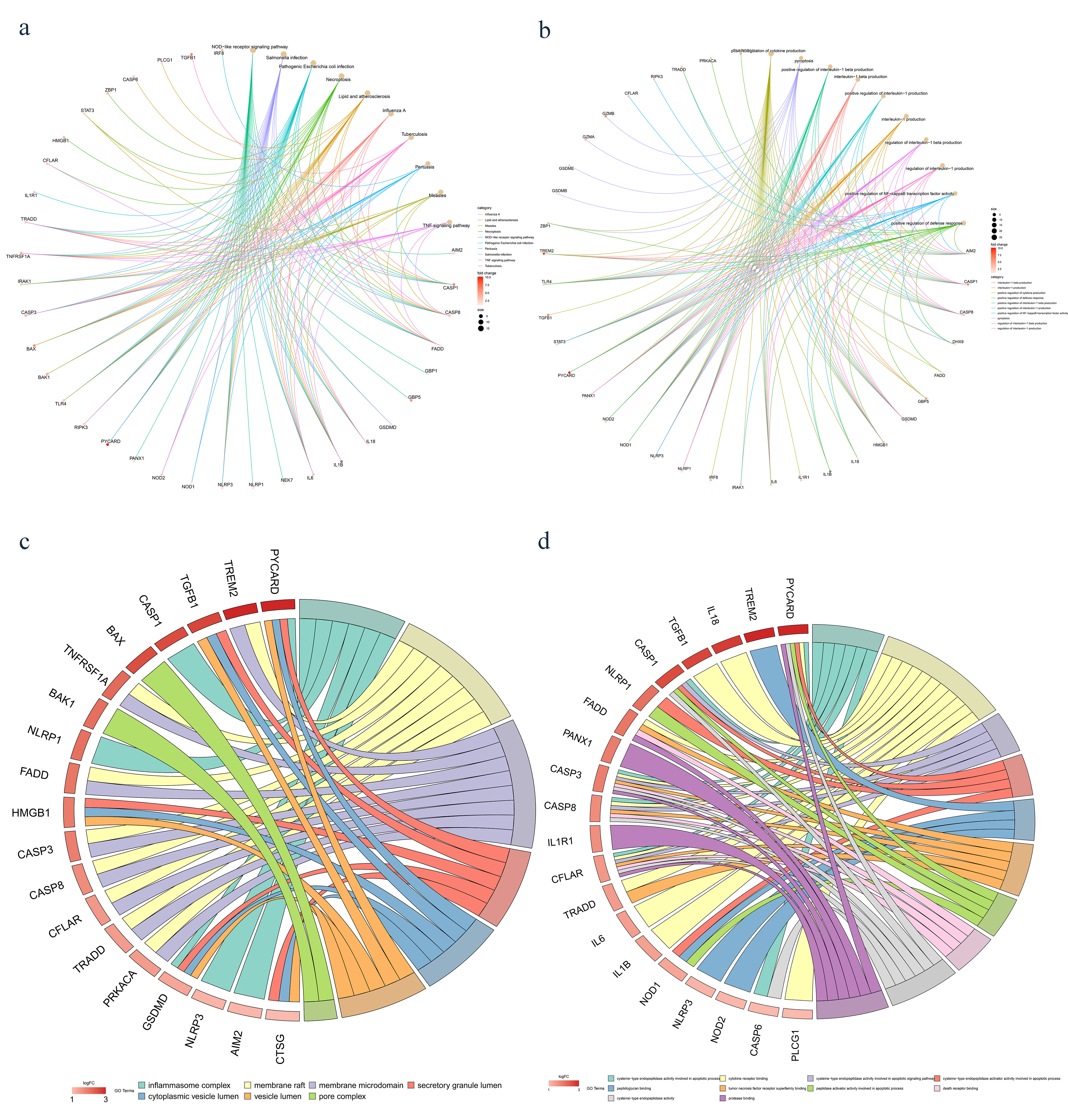

Supplement: Supplementary file 1 [file ijms-23-06178-s001.zip › Figure S2.jpg]

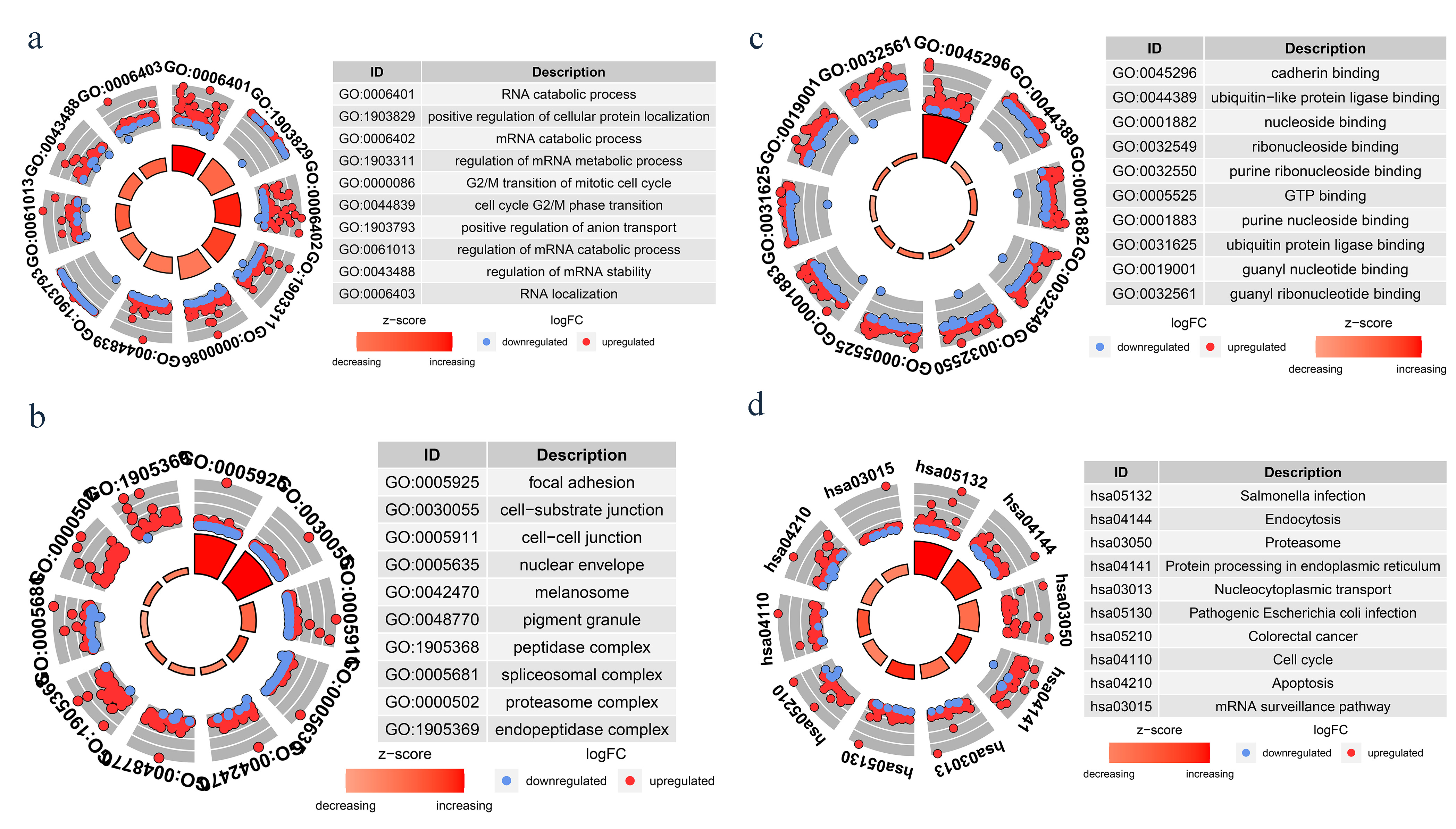

Supplement: Supplementary file 1 [file ijms-23-06178-s001.zip › Figure S3.jpg]

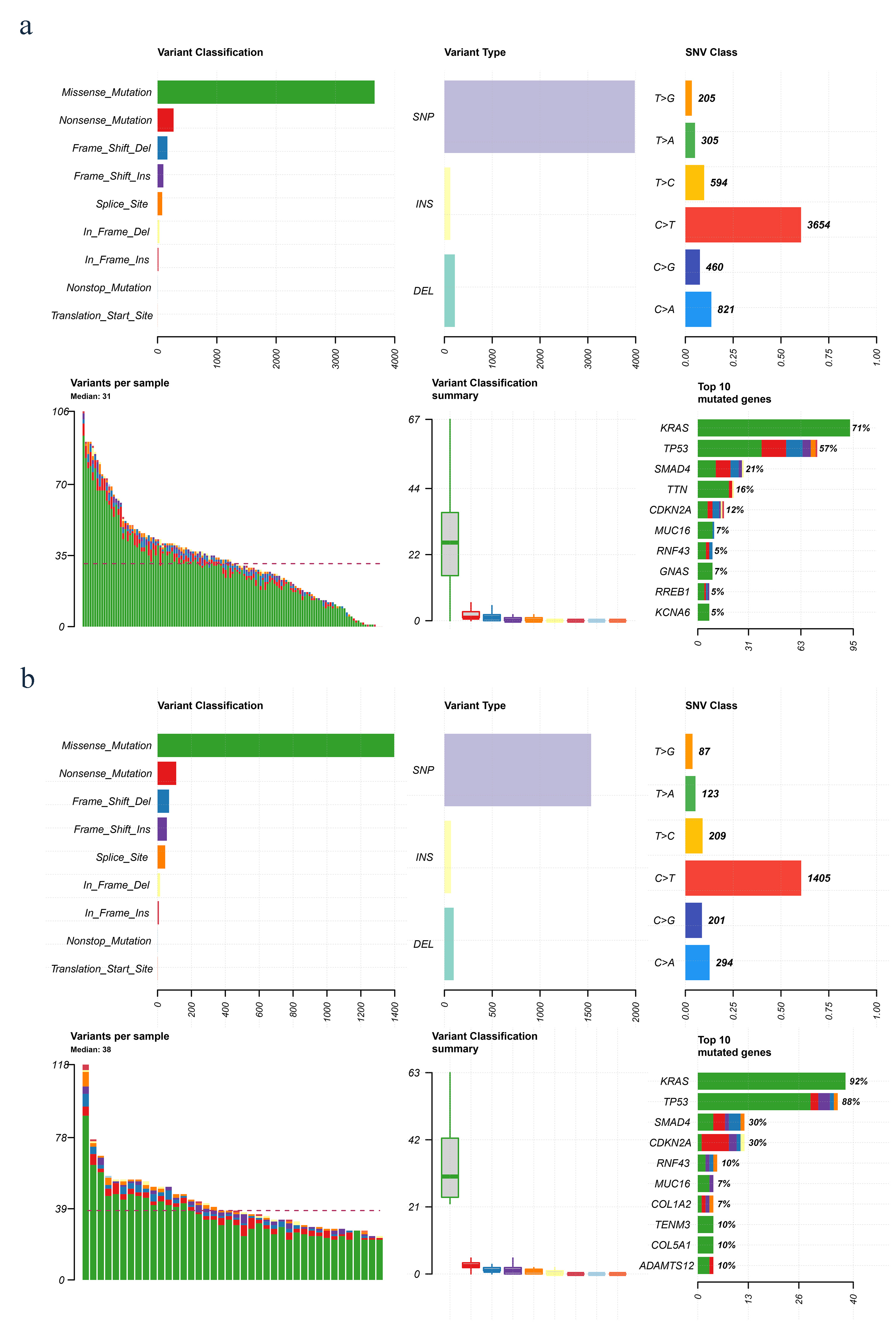

Supplement: Supplementary file 1 [file ijms-23-06178-s001.zip › Figure S4.jpg]

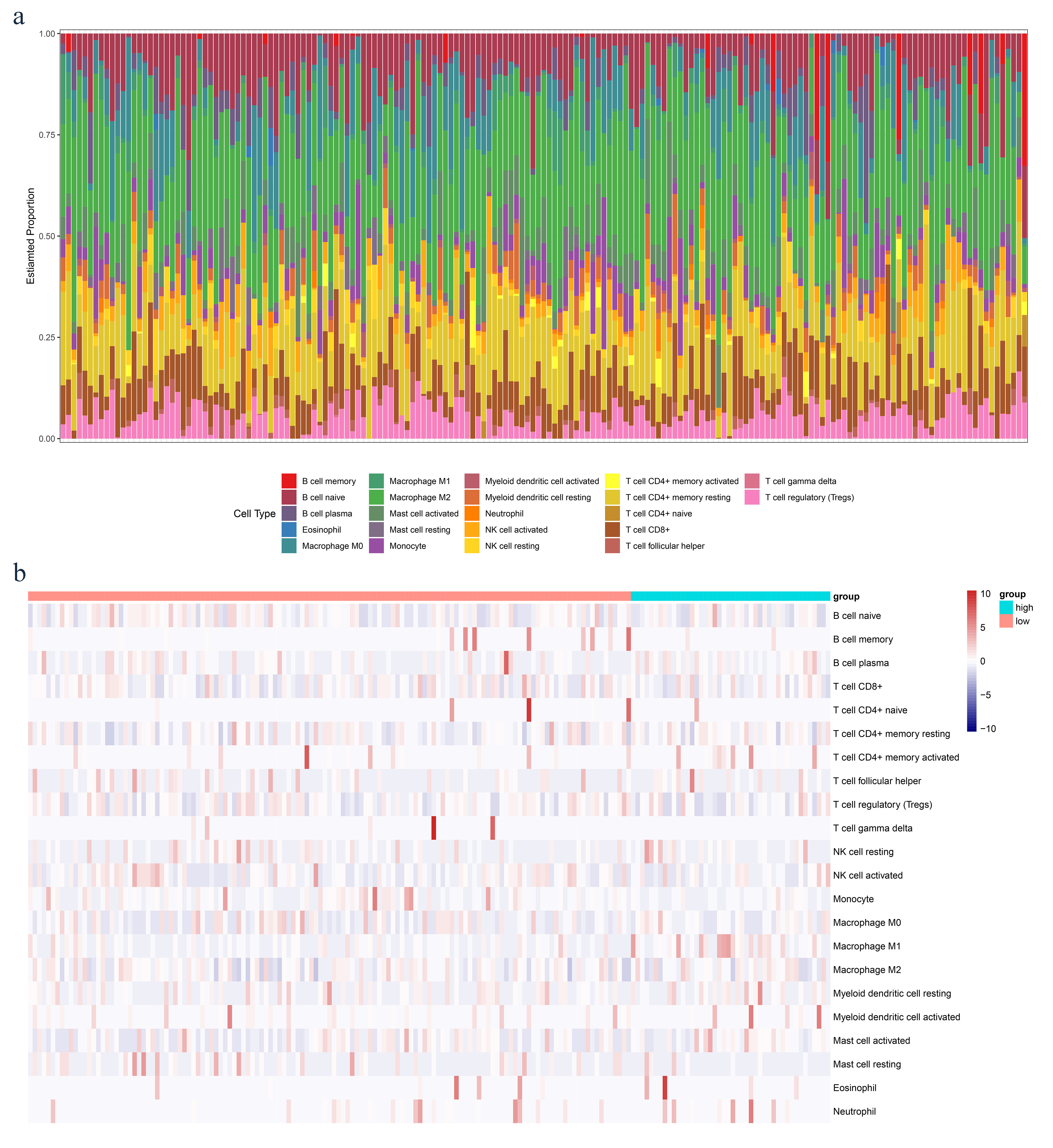

Supplement: Supplementary file 1 [file ijms-23-06178-s001.zip › Figure S5.jpg]

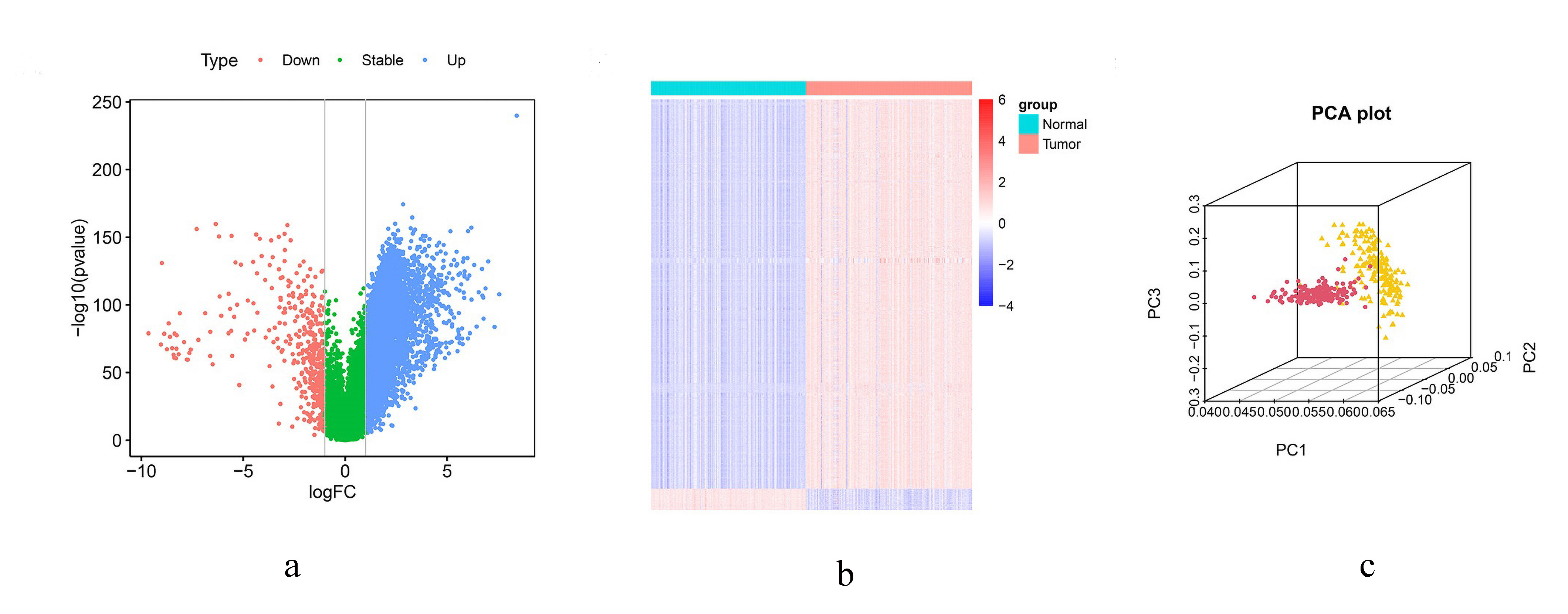

Supplement: Supplementary file 1 [file ijms-23-06178-s001.zip › Figure S1.jpg]
